# Characteristics and Motivational Factors of Whole Blood and Convalescent Plasma Donors during the SARS-CoV-2 Pandemic in Israel

**DOI:** 10.3390/healthcare12050589

**Published:** 2024-03-05

**Authors:** Eilat Shinar, Eli Jaffe, Zvika Orr, Beth G. Zalcman, Joseph Offenbacher, Maxim Quint, Evan Avraham Alpert, Boaz Zadok Weiss, Baruch Berzon

**Affiliations:** 1 Magen David Adom National Blood Services, Ramat Gan 52621, Israel; eilats@mda.org.il; 2 Faculty of Health Sciences, Ben Gurion University of the Negev, Beer Sheva 84105, Israel; 3 Magen David Adom, Tel Aviv 67062, Israel; eliy@mda.org.il (E.J.); maxim.quint@mail.huji.ac.il (M.Q.); 4 Department of Emergency Medicine, Ben Gurion University of the Negev, Beer Sheva 84105, Israel; 5 Selma Jelinek School of Nursing, Jerusalem College of Technology, Jerusalem 91160, Israel; bethz@g.jct.ac.il; 6 Department of Emergency Medicine, New York University Grossman School of Medicine, NYU Langone Health, New York, NY 10016, USA; joseph.offenbacher@nyulangone.org; 7 Department of Emergency Medicine, Hadassah Medical Center-Ein Kerem, Jerusalem 91120, Israel; avraham.alpert@mail.huji.ac.il; 8 Faculty of Medicine, Hebrew University of Jerusalem, Jerusalem 91120, Israel; 9 Department of Emergency Medicine, Shaare Zedek Medical Center, Jerusalem 91031, Israel; bzw@szmc.org.il; 10 Department of Emergency Medicine, Shamir Medical Center, Beer Yaakov 70300, Israel; baruchb@shamir.gov.il

**Keywords:** blood donation, plasma donation, convalescent plasma, COVID-19

## Abstract

Demands for whole blood (WB) and COVID-19 convalescent plasma (CCP) donations during the SARS-CoV-2 (COVID-19) pandemic presented unprecedented challenges for blood services throughout the world. This study aims to understand the motivating factors that drive WB and CCP donations in the context of the pandemic. This cross-sectional study is based on data extracted from surveys of the two volunteer donor cohorts. The findings reveal that when compared to CCP donors, WB donors were more likely to view donation as a form of social engagement (97.7% vs. 87.1%, *p* < 0.01), advantageous in the workplace (46.4% vs. 28.6%, *p* < 0.01), advantageous in their social network (58.6% vs. 47.0%, *p* = 0.01), and view their donation in the context of positive self-satisfaction (99% vs. 95.1%, *p* = 0.01). The average age of CCP donors was 7.1 years younger than those who donated WB (*p* < 0.01). Motivational factors were also analyzed by sex and religiosity. In conclusion, whereas both donor groups showed a high motivation to partake in these life-saving commitments, WB donors were more likely to be motivated by factors that, when better-understood and implemented in policies concerning plasma donations, may help to increase these donations.

## 1. Introduction

Israel’s National Emergency Medical and Blood Services Organization, Magen David Adom (MDA) centralizes the national blood program. Their responsibilities include collecting whole blood (WB), typing, testing, processing into components, storing, and supplying them to all the hospitals in the country, as well as to the military. Magen David Adom Blood Services (MDABS) also operates an active apheresis unit that collects blood components (single-donor plasma and/or platelets) using automated techniques [1]. According to MDABS data, there are approximately 280,000 units of blood and 1000 apheresis donations collected annually, all from volunteer donors [2]. Most blood donors are native-born Israelis and are young males between the ages of 17 and 40. Eighty percent are repeat donors. Most (90%) donors are actively recruited by MDABS in mobile blood drives at schools, places of work, community centers, and military units. Other donors donate at permanent donation sites [3]. These facts are similar to those reported in the United States, where donors are more likely to be younger (18–24 years), US-born, white, and report healthier behaviors [4]. Similarly, in England and North Wales, donations are more common among Whites of British origin, and the median age for a first donation is 28 [5].

Based on previous experience, the use of COVID-19 convalescent plasma (CCP) was introduced by the Israeli Ministry of Health as an optional treatment for patients with moderate or severe COVID-19 to provide temporary “passive immunization” [6]. In March 2020, MDABS was requested to organize the collection, antibody testing, and supply of such units according to the approved national experimental protocol (00830-20-WOMC). Thus, since the outbreak of COVID-19 in Israel, MDABS has been tasked with collecting and supplying both blood units and components and CCP units, while maintaining and augmenting its regular supply to all the hospitals and the military.

Current findings are equivocal on the effect of convalescent plasma administration on the risk of mortality or clinical outcomes. However, there have been findings that suggest that such therapy might reduce disease progression in certain subgroups [7,8,9,10]. A systematic review and meta-analysis of randomized controlled trials found that outpatients with COVID-19 treated with convalescent plasma had a lower risk of requiring hospital care. However, in hospitalized populations, convalescent plasma treatment was not associated with prolonged survival or improved clinical outcomes. These results suggest that this treatment has potential benefits, when given early in the course of the infection, to prevent progression to severe disease [9].

Times of national crisis often place unique challenges on the blood supply due to discrepancies between blood donations and increased demands. However, research is inconclusive as to how this affects blood donations [11].

The first confirmed case of COVID-19 in Israel was on 21 February 2020. This prompted a series of national lockdowns between March 2020 and January 2021. The pandemic presented unprecedented challenges for MDABS, due to a unique coalescence of factors, including an ongoing demand for blood units and components, an evolving need for convalescent plasma, and high levels of societal uncertainty threatening to impact expected patterns of donation [12]. A better understanding of these underlying factors may contribute to efforts to maintain and increase the national blood inventory in response to the health system’s demands during natural or manmade disasters.

Over the years, several studies have been conducted to better understand the characteristics and motivational factors of those who donate blood [13]. It has been recognized that different demographic factors, donor perceptions of the nature of altruism, and societal circumstances affect donation patterns. Studies have found that motivators for blood donation include prosocial activity, which includes altruism, community-based or immediate-circle-based collectivism; the convenience of the physical location of the clinic; personal values such as religious values and personal moral obligation; the perceived need for donation, for example, after a tragic or catastrophic event; indirect reciprocity; marketing efforts; incentives such as money, gifts, a health check, or time off school or work; and social norms. On the other hand, deterrents include inconvenience, ineffective incentives, lack of knowledge surrounding the need for blood donation or locations of blood donation sites, and low involvement [14].

Motivational factors may differ by sociodemographic group. For example, studies have found that donations due to altruistic reasons are weaker among men compared to women, while women were more likely to cite an awareness of the need to donate blood [15]. A meta-analysis of motivators and deterrents among donors found that stratification by donor type, repeat donors, first-time donors, plasma or other blood component donors, past donors, and non-donors indicate different motivators and deterrents of donation. For example, past donors were more likely to point to a perceived need for blood, marketing, and the reputation of the collection agency as motivators. On the other hand, repeat donors and first-time donors were more likely to point to convenience, prosocial motivation, and personal moral values [14].

Furthermore, studies have indicated that motivational differences exist between WB donors and plasma donors. Specifically, as plasma donation is less well-known than blood donation, one study found that plasma donors had higher intentions and self-efficacy compared to blood donors. Additionally, plasma donors were more likely to be less anxious [16]. Another study found that, while blood donors indicated that one of their motivations was that donating blood is relatively easy, plasma donors were motivated by the belief that helping others was in their nature, as well as by taking pride in their donation [17].

Several studies have shown that there were significantly fewer blood donations, in particular, volunteer blood donations, during the pandemic compared to previous years [18,19]. Reported deterring factors include fear and anxiety, lockdowns, and lack of strategies to guarantee the blood donation supply chain [19]. Conversely, a study conducted during the pandemic in the Netherlands found that 80% of donors during a period in 2020 (after the outbreak of the pandemic) reported that they donated blood and blood products to help COVID-19 patients [20].

The purpose of this study was to understand the motivating factors that drive WB and CCP donations in the context of the pandemic, using data extracted from surveys of the two volunteer donor cohorts. Specifically, for WB donors, the aim was to understand what motivated them to leave the safety of their houses during a pandemic to donate blood, especially as at the time of the study, no vaccines were available and most workplaces were closed, which, during non-pandemic times, are popular donation sites.

## 2. Materials and Methods

This was a cross-sectional study conducted over a month (April 2020) in the beginning stages of the pandemic, during the first wave of COVID-19 in Israel. At the time of the survey, there were only a few thousand confirmed cases [21]. Two anonymous surveys were designed, one for WB donors and one for CCP donors. These surveys, including the motivations included in the surveys, were developed by MDABS. A first-draft questionnaire was given to volunteers over the course of two days as a pilot study, and the final questionnaires were developed based on the findings from the pilot. The final surveys included the following:

1. The Blood Donor Motivation survey, which was targeted at both walk-in and invited WB donors across Israel. Respondents were required to be volunteers, meet all the standard blood donors’ criteria [22], be healthy or at least 28 days post convalescence from COVID-19 or exposure to a verified patient. The survey consisted of 25 discrete questions about the participants’ demographic characteristics and motivational factors leading to their WB donation.
2. The Plasma Donor Motivation survey was targeted at convalescent donors, at least 14 days post recovery from COVID-19, who were invited and scheduled to donate plasma collected using automated apheresis equipment for the treatment of COVID-19 patients. The CCP donors were identified based on lists of recovered COVID-19 patients provided to MDABS by the Ministry of Health. MDABS contacted these individuals by telephone and conducted an initial screening to determine if the individual was eligible to donate plasma. If found eligible for donation, the individual was invited to come to a donation site to donate plasma. A secondary screening was conducted at the donation site to rule out any other exclusion criteria prior to donation. In addition to meeting all said standard blood donors’ criteria, CCP donors had to show proof of past illness and recovery from active disease (two negative PCR tests). To mitigate transfusion-related acute lung injury (TRALI), only male donors or nulliparous females were invited to donate CCP. The survey consisted of 39 discrete questions about the participants’ demographic characteristics and motivational factors leading to their CCP donation. Plasma donors were recruited from people who came to donate plasma at the fixed-site donor room located in the MDABS center in Ramat Gan.

The sample included all donors who donated in April 2020, met the said inclusion criteria, and agreed to participate in the study.

### 2.1. Data Collection and Processing

The study was approved by the Scientific Committee of Magen David Adom. Potential respondents were approached a few minutes after completing their blood or plasma donations and were provided with an explanation of the study and its aim. Those who consented to participate were given the relevant survey. The surveys were paper-based and anonymous. They included several question formats: dichotomous, Likert scale, and open-ended.

Results from both surveys were collected and organized into databases using Microsoft Excel (2016). Both databases were established with specific inclusion criteria mandating that: each survey was completed by a participant who met donation eligibility as described above, had included age, sex, and if they were religious or secular in the section on demographic characteristics, and had answered at least one of the seven questions about donation motives. Submitted surveys that did not meet these criteria were excluded from the final database.

The collected sociodemographic information included age, sex, religiosity, and self-reported levels of education. In Israel, a significant portion of the population attends post-high-school-level religious seminaries; this was included as an option for those responding to questions about the level of education. Motivational factors that were collected were based on previous studies as well as on unique characteristics related to blood donation in Israel [13,14,15,17,23,24,25]. These factors included donation as a reflection of religious values, donation as a form of social engagement, donation as positive self-satisfaction, donation as beneficial in the realm of the respondent’s job, and donation as important to individuals close to the respondent and to the public.

### 2.2. Data Analysis

The study was designed to address a specific primary outcome: which, if any, demographic and motivational factors impacted WB vs. CCP donations during the SARS-CoV-2 pandemic. Although the study utilized data from the previously described databases, it was not designed to address all aspects of the original survey, and only data pertaining to the research question were analyzed. Reviewers considered only demographic and motivational factors that were similarly assessed across both donation cohorts and reflecting existing literature about WB donation trends. Ultimately, three demographic and seven motivational characteristics underwent analysis based on these criteria.

All but two aspects of the data analysis were conducted using the R programming language [26]. Potential differences in mean age were determined using a two-sample t test in Google Sheets. Also, summary data were checked in Google Sheets as well as in R Studio. All other comparisons were made using two-sample chi-square proportion tests using R.

## 3. Results

A total of 589 donors met the criteria for inclusion in the final analysis: 284 CCP and 305 WB donors. The age of the CCP donors ranged from 17 to 73 years, with a mean age of 30.4 years. Findings showed that 219 (77.1%) were male, 65 (22.9%) were female. Of the CCP donors, 101 (35.6%) identified as secular and 183 (64.4%) identified as religious.

The WB donor ages ranged between 17 and 68 years with a mean age of 37.5 years. Of the WB donors, 157 were male (51.5%) and 148 were female (48.5%). Of the WB donors, 178 (58.3%) identified as secular and 127 (41.6%) identified as religious (Table 1).

**Figure 1:**
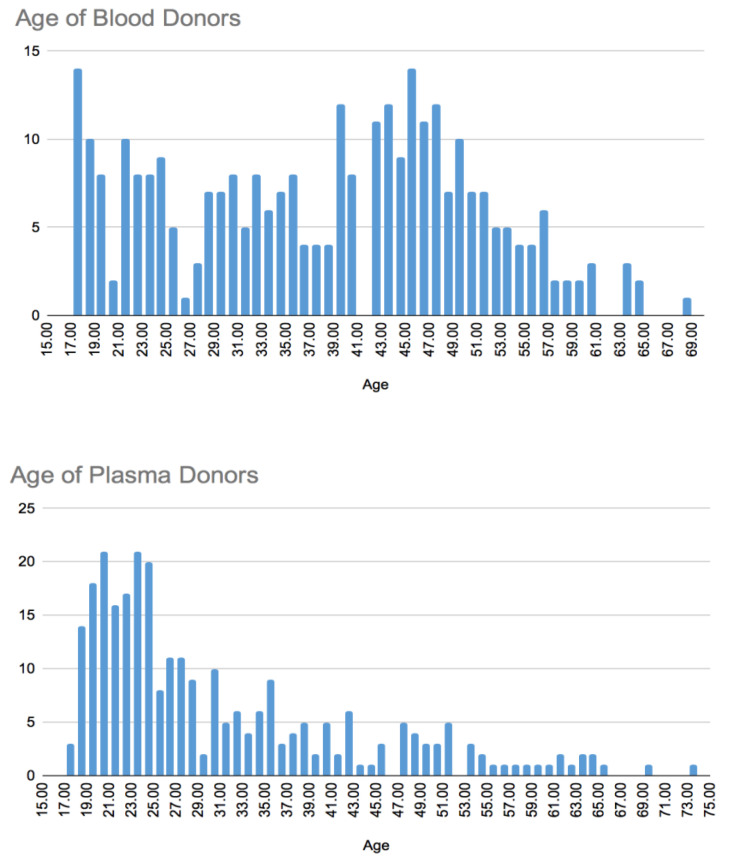
Donor age distribution.

The study’s primary outcome was to identify which, if any, demographic and motivational factors were significantly associated with CCP or WB donations during a defined period of the pandemic. Findings demonstrated that the mean age of WB donors was 7.1 years older than the CCP donors (*p* < 0.01). The WB donor population demonstrated a bimodal distribution, with peak ages in their late teens and early twenties as well as mid-forties. In contrast, the ages of CCP donors demonstrated unimodal distribution with a significantly right-skewed peak in their early twenties (Figure 1).

When compared to CCP donors, WB donors were more likely to view donation as a form of social engagement (97.7% vs. 87.1%, *p* < 0.01), as advantageous in the workplace (46.4% vs. 28.6%, *p* < 0.01) and in their social network (58.6% vs. 47%, *p* = 0.01), and view their donation in the context of positive self-satisfaction (99% vs. 95.1%, *p* = 0.01). No significant differences were found regarding the other factors: donation as important to those close to them (*p* = 0.52), donation as aligning with religious views (*p* = 0.19), and the importance of giving to the public (*p* = 0.44) (Table 2).

Female plasma donors, compared to male plasma donors, were more likely to answer, “The donation is important to those close to me” (*p* = 0.02; −0.12, 0.02), “The donation helps me in my workplace” (*p* < 0.01; 0.05, 0.40), and “The donation helps me in my social networks” (*p* = 0.02; 0.03, 0.40). Differences were also found between female blood donors and female plasma donors as well as between male blood donors and male plasma donors. Female blood donors, compared to female plasma donors, were more likely to answer, “The donation is a way of being socially involved” (*p* < 0.01; 0.01, 0.20). Additionally, female blood donors compared to female plasma donors answered at a higher proportion, “The donation helps me feel good about myself” (*p* = 0.02; −0.01, 0.14). Male blood donors answered, “The donation is a way of being socially involved” at a higher proportion compared to male plasma donors (*p* < 0.01; 0.04, 0.16). Similarly, male blood donors were more likely to answer that, “The donation helps me in my workplace” (*p* < 0.01; 0.15, 0.36) and “The donation helps me in my social networks” (*p* < 0.01; 0.06, 0.29) compared to male plasma donors (Table 3, please see Supplementary Table S1A–D for more information).

Secular blood donors, compared to religious blood donors, were less likely to answer, “The donation is in line with my religious views” (*p* < 0.01; −0.31, −0.14). Similarly, secular plasma donors, compared to religious plasma donors, were less likely to state, “The donation is in line with my religious views” (*p* < 0.01; −0.50, −0.24). Moreover, secular blood donors, compared to religious blood donors, were less likely to state, “The donation helps me in my workplace” and “The donation helps me in my social networks” (*p* = 0.02; −0.26, −0.02).

Secular blood donors, compared to secular plasma donors, were more likely to answer that, “The donation is a way of being socially involved” (*p* < 0.01; 0.02, 0.17). Similarly, religious blood donors, compared to religious plasma donors, were more likely to state, “The donation is a way of being socially involved” (*p* < 0.01; 0.05, 0.18). Additionally, religious blood donors, compared to religious plasma donors, were more likely to report, “The donation helps me in my social networks” (*p* < 0.01; 0.16, 0.36) (Table 4, please see Supplementary Table S2A–D for more information).

WB donors were asked if they knew someone who had COVID-19 before donating, and CCP donors were asked if they knew someone who had severe COVID-19 before donating. Although these questions are not identical and therefore are not directly comparable, the findings show that, among the two groups, most donors did not know someone with COVID-19 before deciding to donate: 91.1% of WB donors (276/303) did not know someone who had COVID-19, and 80.4% of CCP donors (218/271) did not know someone who had severe COVID-19.

## 4. Discussion

Based on previously conducted studies, several well-established motivational factors impacting blood donation have been identified. Furthermore, these factors have been shown to vary based on individual populations’ unique demographic features [14]. Researchers have also considered the impact of broader societal contexts such as mass casualty events [27,28,29]. For example, studies have shown that events, such as the 11 September 2001 attacks in the United States and the 1989 earthquake in San Francisco, impacted donation patterns [27,29]. Another example is the impressive donor response that MDABS has experienced during the war since October 2023.

However, a recent meta-analysis has demonstrated that pandemics and natural disasters impact blood donation trends differently. Specifically, immediately following a natural disaster, there is more likely to be an increase in blood donations compared to a pandemic, during which there is more likely to be a decrease in blood donations. This is primarily due to fear, anxiety, changes in donor acceptance criteria, and lockdowns during a pandemic, which inhibit donors from coming to a clinic to donate blood [19]. However, the motivation behind blood donations during the COVID-19 pandemic stemmed from wanting to help COVID-19 patients [20]. While our study did not compare the rates of blood donation before and during the COVID-19 pandemic, this important context needs to be considered when planning strategies for blood supply during different types of national crises.

In addition, the literature suggests that different types of blood donation are driven by distinct motivational factors. Although the reasons why individuals donate plasma in non-remunerated settings during non-pandemic times are not entirely understood, it has been recognized that differences in perception between plasma and whole blood donation may play a significant role [16,17,30].

Throughout the initial phases of the pandemic, MDABS faced the challenge of maintaining an adequate blood inventory. While there was a decrease in most mobile blood drives due to the closure of workplaces and community centers, followed by national lockdowns, there was no change in the demand for blood by hospitals, who continued their regular activities and elective surgery plans. In addition, there was an unprecedented and rapidly evolving need for convalescent plasma, while high levels of societal uncertainty were threatening to impact expected patterns of donation. It was quickly recognized that barriers to donation that had existed before the COVID-19 outbreak, combined with new pandemic-related stressors, may impose a significant risk for the exacerbation and worsening of existing shortages [31,32,33].

In anticipating these existential challenges, MDABS, like other blood organizations worldwide, sought to develop new strategies to mitigate the impending blood shortage crisis. These strategies included changing the approaches toward blood drive organization in residential neighborhoods to comply with people’s needs for mutual responsibility, while maintaining social distancing and lockdown regulations. Many of these approaches focused on developing a better understanding of potential donor populations and their evolving perceptions towards voluntary blood-product donations in the context of the COVID-19 pandemic [34]. Since the global community has not faced a pandemic of this nature for nearly a century, there are very few existing data on the effects of pandemics on previously described donation trends. However, existing data indicate that the number of blood donations decreases [19].

On-site deferrals in Israel, i.e., the ineligibility of a donor at the site of donation, were lower among plasma donors compared to blood donors. This was due to a pre-donor screening process conducted for plasma donors, which resulted in a relatively healthier cohort. Furthermore, all plasma donors were required to have had a mild or asymptomatic case of COVID-19, as people who had had moderate or severe cases were ineligible [35].

To date, this study is the first to consider the demographic and motivational factors that drive different groups of blood donors in the context of an ongoing pandemic. Uniquely, we looked to consider which, if any, demographic and motivational factors affected convalescent plasma donors (whose product donations were specifically and immediately targeted to the pandemic) compared to whole blood donors during the same period.

The age difference between blood and plasma donation cohorts demonstrated a statistically significant demographic finding. Further, the WB donor population demonstrated a bimodal distribution, whereas the CCP donor population demonstrated a unimodal distribution. The fact that CCP donors were 7.1 years younger than those who donated blood may be related to the fact that younger people with COVID-19 had less symptomatic disease, a lower risk of severe disease and mortality, and their convalescence period was shorter, with fewer sequela [36]. However further research is needed to better explain these findings.

Our sample also had a higher proportion of religious CCP donors compared to secular CCP donors. This may be because ultra-Orthodox Israeli Jews had a high risk of COVID-19 [37,38,39]. By the end of 2020, 42% of all documented cases of COVID-19 in Israel were among ultra-Orthodox individuals, while the ultra-Orthodox population constituted only 12.5% of the Israeli population [37,38]. There are several reasons for this, including many large families in small living spaces, an emphasis on communal gatherings as a way of life, lack of access to reliable and up-to-date information due to cultural restrictions, cultural–religious beliefs and attitudes regarding health-related issues, and a lack of compliance with the Ministry of Health guidelines [40,41,42]. Furthermore, the pandemic broke out in Israel after the holiday of Purim, during which many religious individuals attend synagogues and parties celebrating the holiday. It is believed that this led to increased exposure [43]. Due to a higher rate of infection and a relatively younger population overall, which lowers the risk of severe disease [44], it is possible that a larger number of religious individuals were invited to donate CCP.

Based on a review of the existing literature, we hypothesized that direct personal knowledge of someone needing a donation would be a significant motivating factor for all blood donors. In 2018, Mohammed and Essel found that 90.3% of blood donors were motivated to donate blood when someone they knew needed blood [45]. In contrast, this study demonstrates that only 8.9% of blood donors knew someone who had COVID-19 before donating, and 19.6% of convalescent plasma donors knew someone who had severe COVID-19 before donating. These findings support our previous knowledge that, in contrast to traditional donation patterns, the motivation among Israeli donors is different from other countries with regard to personal knowledge of someone requiring a donation. Both blood and plasma donations are perceived as a means of social engagement and a form of positive self-satisfaction, and more so during the pandemic. Of particular interest, both blood donation (generally not seen as a specific therapy for COVID-19 patients) and plasma donation (a product very specifically associated with COVID-19 therapy) deviated from expected motivational factors as described in the pre-pandemic literature. However, compared to WB donors, more CCP donors knew someone with COVID-19 before donation.

Blood donors were more likely than plasma donors to report that donation was beneficial to them in relation to their job. It is known, that if workers are given an incentive to donate blood, this increases the chances of donation [46]. Some places of employment encourage their workers to engage in prosocial activities as part of the employee experience. Blood donation is one such activity. However, many people think of whole blood donation when they think about blood donation; therefore, it may be that since plasma donation is lesser-known and takes more effort, fewer people are likely to engage in plasma donation within the context of their jobs [16,17]. A similar explanation can be suggested for the finding that blood donors were more likely than plasma donors to view donation as advantageous in their social network: the donors’ social environments are more familiar with WB donations than with CCP donations. Furthermore, plasma donors have been found to have higher intentions, being driven more by benevolence [17]. Therefore, it is more likely that someone who donates plasma donates with benevolent intentions, and not merely to engage in accepted social activities [17]. These findings call for a policy that would increase the public understanding and awareness of the meaning of, and need for, plasma donations.

Interestingly, almost all blood donors (97.7%) affirmatively answered that “The donation is a way of being socially involved”, compared to 87.1% of plasma donors. This difference exists also when sub-setting by gender and religiosity. As social engagement is how a person connects with their community, this significant difference may be related to differences in the way blood donation and plasma donation are regarded. This was noted in a study conducted by Delépine-Farvacques et al. [47], which found that people were more likely to donate plasma when they were specifically requested to donate. This may be due to the amount of time it takes to donate plasma in comparison to whole blood [48].

Religious blood and plasma donors reported that donation was in line with their religious views. Furthermore, religious blood donors were more likely than secular blood donors to view donation as advantageous in their workplace and their social networks. These findings are in keeping with previous studies that have investigated a link between religious observance and participation in blood donation [49,50,51]. Religion acts as both a social network and belief system, promoting the idea of performing good deeds and helping others [52]. Messages related to blood donation as a way to do a good deed and help others are often relayed by religious leaders and have been found to increase blood donation rates among followers [49]. A study in Bangladesh found that, among people who consider blood donation a religious act, the chance of becoming a regular blood donor was twice as high [50]. In a country like Israel, with a large religious population, the religiosity-related motivational factors can be leveraged to encourage both blood and plasma donations.

There are several limitations to this study that should be addressed. First, the study focused solely on the motivational aspects of donating WB or CCP during the pandemic, not on the effectiveness of treatment or outcomes of the donated components. Additionally, no information was collected regarding previous blood donor experience and motivations, limiting the ability to compare current motivations with pre-pandemic motivations. The data collected only examined individuals who chose to donate with a bias toward self-selecting participants. Furthermore, the surveys relied solely on self-reporting, allowing room for response biases.

## 5. Conclusions

Pre-pandemic literature suggested that altruism and other related motivational factors strongly tied to beneficence were significant in describing blood donation patterns. This study found that when comparing blood to convalescent plasma donation cohorts, those who donated WB were more likely to view their donation as a form of social engagement, as being more advantageous in their workplace and social network, and were ultimately more likely to describe their donation as a form of positive self-satisfaction. These findings seem to strongly suggest that, in a country where solidarity and lifesaving by blood donation are highly valued, the pandemic only emphasized them, having an interesting impact on donations not previously described in the pre-COVID literature. Implementing the new understanding of the motivational factors in future public policies concerning plasma donations may contribute to encouraging and increasing these donations.

## Figures and Tables

**Table 1:** Donor demographic data.

Demographic Variables	Blood Donors (%)	Plasma Donors (%)	*p*-Value	C.I. (95%)
Sample Size	305	284		
Age				
Mean Age (years)	37.5	30.4	*p* < 0.01	
Age Range (years)	17–68	17–73		
Sex			*p* < 0.01	−0.33, −0.18
Male	157 (51.5)	219 (77.1)		
Female	148 (48.5)	65 (22.9)		
Religious or Secular			*p* < 0.01	−0.29, −0.12
Religious	127 (41.6)	183 (64.4)		
Secular	178 (58.3)	101 (35.6)		

**Table 2:** Analysis of donor motivation by donor type.

Motivational Factors	Blood Donors (%)	Plasma Donors (%)	*p*-Value	C.I. (95%)
I believe that giving to the public is important	305 (100.0)	275 (99.3)	0.44	−0.01, 0.02
The donation is in line with my religious views	230 (80.1)	225 (85.6)	0.19	−0.11, 0.02
The donation is a way of being socially involved	292 (97.7)	223 (87.1)	<0.01	−0.06, 0.15
The donation is important to those close to me	275 (93.2)	222 (91.4)	0.52	−0.03, 0.07
The donation helps me feel good about myself	299 (99.0)	252 (95.1)	0.01	0.01, 0.07
The donation helps me in my workplace	135 (46.4)	65 (28.6)	<0.01	0.09, 0.26
The donation helps me in my social networks	173 (58.6)	109 (47.0)	0.01	0.03, 0.26

**Table 3:** Analysis of donor motivation by gender.

Motivational Factors	Female Blood Donors (%)	Male Blood Donors (%)	Female Plasma Donors (%)	Male Plasma Donors (%)
I believe that giving to the public is important	148 (100.0)	157 (100.0)	64 (98.5)	211 (99.5)
The donation is in line with my religious views	110 (81.5)	120 (85.5)	47 (81.0)	178 (86.8)
The donation is a way of being socially involved	142 (98.6)	150 (96.8)	53 (88.3)	170 (86.7)
The donation is important to those close to me	130 (91.5)	145 (94.8)	54 (94.7)	168 (90.3)
The donation helps me feel good about myself	145 (99.3)	154 (98.7)	62 (92.5)	190 (95.0)
The donation helps me in my workplace	60 (43.5)	75 (49.0)	24 (44.4)	41(23.7)
The donation helps me in my social networks	81 (57.4)	92 (59.7)	34 (61.8)	75 (42.4)

**Table 4:** Analysis of donor motivation by level of observance.

Motivational Factors	Secular Blood Donors (%)	Religious Blood Donors (%)	Secular Plasma Donors (%)	Religious Plasma Donors (%)
I believe that giving to the public is important	178 (100.0)	127 (100.0)	98 (99.0)	177 (99.4)
The donation is in line with my religious views	113 (71.1)	117 (93.6)	50 (61.0)	175 (96.7)
The donation is a way of being socially involved	170 (97.7)	122 (97.6)	84 (88.4)	139 (86.3)
The donation is important to those close to me	158 (91.9)	117 (95.1)	79 (74.0)	143 (89.9)
The donation helps me feel good about myself	177 (99.4)	122 (98.4)	94 (96.9)	158 (94.0)
The donation helps me in my workplace	68 (40.5)	67 (54.5)	50 (47.6)	80 (42.8)
The donation helps me in my social networks	91 (52.9)	82 (66.7)	46 (54.1)	63 (42.9)

## Data Availability

Data are available upon reasonable request.

## Supplementary Materials

The following supporting information can be downloaded at: https://www.mdpi.com/article/10.3390/healthcare12050589/s1, Table S1: (A) Blood Donors by Gender, (B) Plasma Donors by Gender, (C) Female Donors, (D) Male Donors; Table S2: (A) Blood Donors by Religiosity, (B) Plasma Donors by Religiosity, (C) Secular Donors, (D) Religious Donors.
