# Supplementary material for: Characteristics and Motivational Factors of Whole Blood and Convalescent Plasma Donors during the SARS-CoV-2 Pandemic in Israel
Source: Healthcare (Basel). 2024 Mar 5;12(5):589. doi: 10.3390/healthcare12050589 (PMC10931588; doi:10.3390/healthcare12050589)
Supplement: Supplementary file 1 [file healthcare-12-00589-s001.zip › healthcare-2813737-supplementary.pdf]

Supplementary Tables

Table S1A: Blood Donors by Gender

Motivational Factors	Female Blood Donors	Male Blood Donors	P Value	95% Confidence Interval
I believe that giving to the public is important	148/148	157/157	P = 1.00	0.00: 0.00
The donation is in line with my religious views	110/135	120/149	P = 0.96	-0.09: 0.12
The donation is a way of being socially involved	142/144	150/155	P = 0.50	-0.02: 0.60
The donation is important to those close to me	130/142	145/153	P = 0.38	-0.10: 0.03
The donation helps me feel good about myself	145/146	154/156	P = 1.00	-0.02: 0.03
The donation helps me in my workplace	60/138	75/153	P = 0.40	-0.18: 0.07
The donation helps me in my social networks	81/141	92/154	P = 0.78	-0.14: 0.10

Table S1B: Plasma Donors by Gender

Motivational Factors	Female Plasma Donors	Male Plasma Donors	P Value	95% Confidence Interval
I believe that giving to the public is important	64/65	211/212	P = 0.96	-0.05: 0.03
The donation is in line with my religious views	47/58	178/205	P = 0.37	-0.18: 0.06
The donation is a way of being socially involved	53/60	170/196	P = 0.92	-0.08: 0.12
The donation is important to those close to me	54/57	168/186	P = 0.02	-0.12: 0.02
The donation helps me feel good about myself	62/67	190/200	P = 0.65	-0.10: 0.06
The donation helps me in my workplace	24/54	41/173	P < 0.01	0.05: 0.40
The donation helps me in my social networks	34/55	75/177	P = 0.02	0.03: 0.40

Table S1C: Female Donors

Motivational Factors	Female Blood Donors	Female Plasma Donors	P Value	95% Confidence Interval
I believe that giving to the public is important	148/148	64/65	P = 0.67	-0.03: 0.06
The donation is in line with my religious views	110/135	47/58	P = 1.00	-0.12: 0.12
The donation is a way of being socially involved	142/144	53/60	P < 0.01	0.01: 0.20
The donation is important to those close to me	130/142	54/57	P = 0.64	-0.11: 0.05
The donation helps me feel good about myself	145/146	62/67	P = 0.02	-0.01: 0.14
The donation helps me in my workplace	60/138	24/54	P = 1.00	-0.18: 0.16
The donation helps me in my social networks	81/141	34/55	P = 0.69	-0.21: 0.12

Table S1D: Male Donors

Motivational Factors	Male Blood Donors	Male Plasma Donors	P Value	95% Confidence Interval
I believe that giving to the public is important	157/157	211/212	P = 1.00	-0.01: 0.02
The donation is in line with my religious views	120/149	178/205	P = 0.15	-0.15: 0.02
The donation is a way of being socially involved	150/155	170/196	P < 0.01	0.04: 0.16
The donation is important to those close to me	145/153	168/186	P = 0.18	-0.02: 0.12
The donation helps me feel good about myself	154/156	190/200	P = 0.10	-0.00: 0.08
The donation helps me in my workplace	75/153	41/173	P < 0.01	0.15: 0.36
The donation helps me in my social networks	92/154	75/177	P < 0.01	0.06: 0.29

Table S2A: Blood Donors by Religiosity

Motivational Factors	Secular Blood Donors	Religious Blood Donors	P Value	95% Confidence Interval
I believe that giving to the public is important	178/178	127/127	P = 1.00	0.00: 0.00
The donation is in line with my religious views	113/159	117/125	P < 0.01	-0.31: -0.14
The donation is a way of being socially involved	170/174	122/125	P = 1.00	-0.03: 0.04
The donation is important to those close to me	158/172	117/123	P = 0.3877	-0.10: 0.03
The donation helps me feel good about myself	177/178	122/124	P = 0.75	-0.02: 0.04
The donation helps me in my workplace	68/168	67/123	P = 0.02	-0.26: -0.02
The donation helps me in my social networks	91/172	82/123	P = 0.02	-0.26: -0.02

Table S2B: Plasma Donors by Religiosity

Motivational Factors	Secular Plasma Donors	Religious Plasma Donors	P Value	95% Confidence Interval
I believe that giving to the public is important	98/99	177/178	P = 1.00	-0.03: 0.02
The donation is in line with my religious views	50/82	175/181	P < 0.01	-0.50: -0.24
The donation is a way of being socially involved	84/95	139/161	P = 0.77	-0.07: 0.11
The donation is important to those close to me	79/84	143/159	P = 0.40	-0.04: 0.12
The donation helps me feel good about myself	94/97	158/168	P = 0.45	-0.03: 0.09
The donation helps me in my workplace	50/105	80/187	P = 0.50	-0.08: 0.17
The donation helps me in my social networks	46/85	63/147	P = 0.12	-0.03: 0.25

Table S2C: Secular Donors

Motivational Factors	Secular Blood Donors	Secular Plasma Donors	P Value	95% Confidence Interval
I believe that giving to the public is important	178/178	98/99	P = 0.77	-0.02: 0.04
The donation is in line with my religious views	113/159	50/82	P = 0.15	-0.04: 0.24
The donation is a way of being socially involved	170/174	84/95	P < 0.01	0.02: 0.17
The donation is important to those close to me	158/172	79/84	P = 0.70	-0.10: 0.05
The donation helps me feel good about myself	177/178	94/97	P = 0.25	-0.02: 0.07
The donation helps me in my workplace	68/168	50/105	P = 0.30	0.15: 0.40
The donation helps me in my social networks	91/172	46/85	P = 0.96	-0.15: 0.13

Table S2D: Religious Donors

Motivational Factors	Religious Blood Donors	Religious Plasma Donors	P Value	95% Confidence Interval
I believe that giving to the public is important	127/127	177/178	P = 1.00	-0.01: 0.02
The donation is in line with my religious views	117/125	175/181	P = 0.32	-0.09: 0.03
The donation is a way of being socially involved	122/125	139/161	P < 0.01	0.05: 0.18
The donation is important to those close to me	117/123	143/159	P = 0.17	-0.02: 0.12
The donation helps me feel good about myself	122/124	158/168	P = 0.12	-0.01: 0.09
The donation helps me in my workplace	67/123	80/187	P = 0.06	-0.00: 0.24
The donation helps me in my social networks	82/123	63/147	P < 0.01	0.16: 0.36
